# Is vulnerability to cardiometabolic disease in Indians mediated by abdominal adiposity or higher body adiposity

**DOI:** 10.1186/1471-2458-14-1239

**Published:** 2014-12-01

**Authors:** Hannah Kuper, Amy Taylor, Kankipati Vijay Radha Krishna, Yoav Ben-Shlomo, Ruby Gupta, Bharati Kulkarni, Dorairaj Prabhakaran, George Davey Smith, Jonathan Wells, Shah Ebrahim, Sanjay Kinra

**Affiliations:** Clinical Research Department, London School of Hygiene & Tropical Medicine, Keppel Street, London, WC1E 7HT UK; School of Experimental Psychology, University of Bristol, Bristol, UK; National Institute of Nutrition, Hyderabad, India; School of Social and Community Medicine, University of Bristol, Bristol, UK; Centre for Chronic Disease Control, New Delhi, India; Childhood Nutrition Research Centre, UCL Institute of Child Health, London, UK; Department of Non Communicable Disease Epidemiology, London School of Hygiene & Tropical Medicine, London, UK

**Keywords:** Adiposity, India, Cardiometabolic, Diabetes, Hypertension, Cardiovascular, Dual-energy X-ray absorptiometry

## Abstract

**Background:**

Indians may be particularly vulnerable to cardiometabolic disease, potentially due to higher body fat for a given BMI, or a tendency towards depositing abdominal adiposity. The aim of the study is to assess whether different measures of the distribution of adiposity (abdominal versus whole body) or amount of adiposity (DXA versus traditional anthropometric) are better at predicting prevalent cardiometabolic risk markers in an Indian population.

**Methods:**

Participants were recruited from the Indian Migration Study (IMS) and the Andhra Pradesh Children and Parent Study (APCAPS). Participants attended a clinic in Hyderabad, India, January 2009-December 2010. Adiposity was measured by conventional anthropometry (including weight, height, waist) and DXA scanning (whole body and abdominal). Blood samples were taken and assessed for fasting plasma glucose, insulin, cholesterol, and triglycerides and blood pressure was measured. Lifestyle data were collected by questionnaire.

**Results:**

We invited 4 617 participants to the clinic (1 995 IMS; 2 622 APCAPS) and examined 918 from IMS (46%) and 1 451 from APCAPS (55%). There were strong and consistent relationships between adiposity and cardiometabolic risk factors. Cardiometabolic risk factors did not appear to be more strongly associated with DXA measures as opposed to BMI, or skinfold measures of body fat. There was some evidence that WHR was more closely related to diabetes than total body adiposity, but this was not apparent for the other measures of abdominal adiposity (DXA measures, waist circumference) or other cardiometablic risk factors.

**Conclusions:**

No strong evidence supports that DXA measures or abdominal measures of adiposity are better at predicting the prevalence of cardiometabolic risk factors in comparison to BMI.

**Electronic supplementary material:**

The online version of this article (doi:10.1186/1471-2458-14-1239) contains supplementary material, which is available to authorized users.

## Background

India is experiencing a rapid increase in the prevalence of obesity, type 2 diabetes and deaths from cardiovascular disease (CVD) [1–4]. This epidemic has been attributed to rising urbanisation [5, 6], and consequent changes in patterns of diet [7] and physical activity [8]. Asian countries have similar prevalence of diabetes to Western countries, despite lower obesity as measured through Body Mass Index (BMI) [9]. These ecological data are supported by observational studies, which suggest that diabetes develops at lower levels of BMI among Asians compared to Whites [10, 11]. Furthermore, several studies suggest that Indian men are less insulin sensitive than Whites regardless of their BMI [12], with insulin resistance occurring even in lean Indians [13, 14] and Indian adolescents [15].

There are several potential explanations for the vulnerability among Indians to diabetes and other cardiometabolic diseases. Indians may have higher percentage body fat for a given BMI compared with White populations [12, 14, 16, 17], even as newborns [18]. Alternatively, the vulnerability may result from a predisposition towards abdominal adiposity [12–14, 19], which may be more closely linked to risk of cardiometabolic disease than more general obesity measures [20–22]. A third explanation argues for vulnerability towards diabetes and other chronic disease among people of Indian descent which is not related to adiposity [3, 9, 23], including genetic variants, though this is still strongly debated [24–29].

The key to unraveling this question is to obtain an accurate measure of adiposity and abdominal adiposity in an Indian population [30]. Low-technology methods, such as BMI measurement, may not correspond well to adiposity, as they are also dependent on the amount of lean mass and height [17]. Waist-hip ratio (WHR) and skinfold thickness may be difficult to measure reliably and hence have a greater degree of non differential misclassification which would attenuate associations [30]. Dual-energy X-ray absorptiometry (DXA) scanning provides precise measures of the amount and distribution of fat tissue, including abdominal adiposity, through the differential absorption of X rays of two different energies. However, the use of DXA scanning in large-scale population studies adds substantially to costs and complexity so is not feasible in many settings.

The aim of the study is to assess whether different measures of the distribution of adiposity (abdominal versus whole body) or amount of adiposity (DXA versus traditional anthropometric) are better at predicting prevalent cardiometabolic risk markers in an Indian population.

## Methods

### Study population

The current analyses were based on cross-sectional data obtained during clinical investigations of participants from two previously studied cohorts living in the city of Hyderabad, India, and its surrounding areas.

The Hyderabad arm of the Indian Migration Study (IMS) is a cohort study constituted of rural to urban migrants and their spouses recruited from a factory in Hyderabad and their siblings who had remained in a rural area [6]. As a control group, factory workers born in urban areas and their urban siblings were also recruited. The baseline was conducted between 2005 and 2007, during which time 1 995 participants were examined in Hyderabad (overall response rate for IMS 50%).

The Andhra Pradesh Children and Parent Study (APCAPS) is based on the Hyderabad Nutrition Trial (HNT) conducted in 1987-1990 [31]. The HNT consisted of 2 622 children who were born in 29 villages around Hyderabad between 1987 and 1990. In half of the villages, pregnant women and their offspring received food supplementation, providing 2.51 MJ and 20 g protein to the women and half this amount to the children. In the control villages they received no food supplementation. Between 2003 and 2005, 1165 of the children attended a research clinic [32]. These children, their parents and siblings now form the APCAPS cohort.

The participants of these two cohorts were invited to attend a clinic at the National Institute of Nutrition between January 2009 and December 2010.

### Data collection

#### Measures of adiposity

##### DXA scanning

Participants underwent whole body dual energy X-ray absorptiometry (DXA) scans on a Hologic DXA machine (Discovery A model, 91% of scans) or a Hologic QDR 4500 Elite machine (9% of scans).

The whole body scan was performed with the participant supine on the scanning bed with their arms resting by their sides to provide measures of total body fat (g) and total body percentage fat (%).The Hologic software also defined the trunk region, with manual adjustment by a single DXA technician where necessary. Abdominal body composition measures were calculated for two regions of interest: L1-L4 and L2-L4. These regions of interest were drawn twice on to the whole body scan using the Hologic software by the same technician and the average estimate of the two measurements was used in the analyses.

Whole body scans were visually checked for artifacts and those with major artifacts (e.g. movement artifacts) were removed from the analyses. For quality assurance, a spine phantom was scanned every day to check for acceptable ranges.

##### Anthropometric data

Weight was measured to the nearest 0.1 kg with digital Seca weighing machine (http://www.seca.com) and standing height to the nearest 1 mm with a plastic stadiometer (Leicester height measure; supplied by Chasmors, London). Waist circumference (WC) was measured to the nearest mm using a non-stretch metal tape at the narrowest point of the abdomen between the ribs and the iliac crest at the end of expiration and hip circumference at the widest part of the buttock. Each measure was assessed twice, and the average was used in the analysis. We measured skinfold thickness at five sites (biceps, triceps, subscapular, suprailiac and calf) in triplicate to the nearest 0.2 mm using the Holtain calliper (Holtain, Dyfed, UK) and used the average in the analysis to calculate percent body fat with the Durnin and Womersley formula, as it was most widely used [33].

##### Laboratory assessment

Participants were asked to attend fasting and the time of the last meal was recorded. Venous blood samples (20 mL) were collected. IMS participants underwent a standard glucose tolerance test, unless they were diabetic or pregnant, and a second blood sample was taken after two hours.

Blood samples, with the exception of glucose assays, were separated and stored at -20 °C locally and transported to the Centre for Chronic Disease Control laboratory, New Delhi for lipid and insulin analysis. Serum HDL cholesterol was estimated directly by an elimination method, total cholesterol by an enzymatic endpoint method, and triglycerides by GPO-PAP method. Insulin was assessed by ELISA method using kits from MERCODIA (Uppsala, Sweden). The quality of local assays was checked with regular external standards and internal duplicate assays and monitored by All India Institute of Medical Sciences (AIIMS). The Cardiac Biochemistry Lab, AIIMS, is part of the UK National External Quality Assessment (http://www.ukneqas.org.uk) programme and External Quality Assessment Scheme from RANDOX for quality assurance of Insulin and biochemical assays respectively. Glucose was measured on the day of the sample collection at the National Institute of Nutrition with the GOD-PAP method using RANDOX kits.

##### Cardiovascular measures

We used a validated oscillometric device (OMRON M5-; Omron, Matsusaka Co, Japan) to measure systolic blood pressure (SBP) and diastolic blood pressure (DBP) in the sitting position on the right arm using an appropriate sized cuff after a period of 5 minutes rest. We took three measurements, 2-3 minutes apart, and used the average of the last two measurements for analysis.

##### Questionnaire data

Participants were interviewed using a structured questionnaire. Socioeconomic position was assessed using a subset of 14 of 29 questions from the Standard of Living Index (SLI), where a higher score denotes higher socioeconomic position. Physical activity over the past week was assessed based on the average amount of time and frequency of participation in different activities. Diet was measured with a food frequency questionnaire. Information was also collected about tobacco use, alcohol consumption and medical history.

### Training and pilot testing

Training of fieldworkers and screening staff was conducted over a two week period and repeated at the mid-point of the study, which included standardization of the anthropometrists against a gold standard and assessment of within and between observer repeatability. A pilot study was conducted over a one week period.

### Statistical analyses

#### Outcome measures

A diagnosis of diabetes was made using the World Health Organization fasting plasma glucose criterion of ≥7.0 mmol/l or 2 hour post glucose load ≥11.1 mmol/l [34], or self report of a diagnosis of diabetes. Impaired fasting glucose (IFG) was defined by fasting plasma glucose criterion of 6.1-6.9 mmol/l and 2 hour post glucose load <7.8 mmol/l. Insulin resistance was defined as being in the top quartile of Homeostasis Model Assessment (HOMA), excluding diabetic individuals and those who had not fasted [35]. Hypertension was defined through self report or blood pressure ≥140/90 mm Hg.

#### Measures of adiposity

Six measures were used in analyses: DXA measures of i) total body and ii) abdominal adiposity, anthropometric measures of iii) BMI, iv) waist-hip ratio, v) waist circumference and vi) body fat based on skinfold thickness (triceps and subscapular) [33].

#### Covariates

Metabolic equivalent tasks (METs) and dietary energy intake were estimated from the questionnaire. We derived an socio-economic status score by weighting the SLI question items to give a maximum score of 38, using standard weights [36].

All analyses were conducted in Stata (version 12). All analyses were performed stratified by study. To allow comparison between different anthropometric measures, all measures were converted to sex and study specific z-scores. Associations with continuously measured outcomes were investigated using multivariable linear regression. Multivariable logistic regression was undertaken for categorical outcomes (diabetes, IFG, insulin resistance, hypertension), and these analyses were restricted to IMS participants because of the very low number of cases among the APCAPS subjects. Outcome measures which were right skewed were log transformed and results from regression analyses of these variables are presented as ratios of geometric means. Regression analyses were adjusted for age, sex, smoking, physical activity, socioeconomic class, alcohol consumption and dietary energy intake. Age was treated as a continuous variable in the APCAPS study but as a categorical variable in the IMS (<40, 40-49, 50-59, 60+ years) because there was evidence that the association between age and adiposity was not linear. DXA measures were also adjusted for DXA machine, DXA total fat (grams) and DXA L1-L4 were additionally adjusted for height. Robust standard errors were calculated to account for siblings within the dataset in both cohorts. Analyses were based on subjects with complete anthropometric and covariate data. Men and women were analysed together, but we tested for sex interactions using Likelihood Ratio Tests. Evidence for statistical heterogeneity in effect estimates between different measures of adiposity and outcome measures was assessed by the I^2^ statistic using the “metan” command in Stata.

### Ethical approval and consent

Ethical approval was obtained from the AIIMS, the National Institute of Nutrition and the London School of Hygiene & Tropical Medicine. Consent was sought from the community leaders in the villages for the APCAPS study, and from the factory managers for the Indian Migrant Study. Informed written consent was collected from all participants. All participants diagnosed with potential medical conditions were referred for appropriate management.

## Results

We invited 4 617 people (1 995 from IMS and 2 622 from APCAPS) to attend the clinic at the National Institute of Nutrition and examined 2369 (51%), 918 from IMS (46%) and 1 451 from APCAPS (55%). Among the IMS subjects, clinic attendees did not differ in age from non attendees (47.8 vs 47.2 years, *P* = 0.15) or by percentage female (47% vs 48%, *P* = 0.82). A higher proportion of clinic attendees were urban dwellers (61% vs 47%, *P* <0.001). Among the APCAPS participants, clinic attendees were similar in age to non attendees (20.1 vs 20.2 years, *P* = 0.03) but were much more likely to be male (68% vs 28%, *P* <0.001).

The APCAPS participants were younger than those from the IMS (Hyderabad) sample, and had generally lower SLI (Table 1). Levels of physical activity and total caloric energy intake were higher in the APCAPS sample compared with IMS. Smoking and alcohol consumption were very rare among females from both the APCAPS and IMS groups. Among men, the prevalence of ever smoking was higher among the IMS subjects compared with the APCAPS subjects, while the levels of alcohol consumption were similar in the two groups and relatively modest. All measures of adiposity, were substantially higher among the IMS participants compared to those from APCAPS as were glucose, insulin, triglycerides and cholesterol.

**Table 1:** **Descriptive data for total population (N = 2160)**

	IMS		APCAPS	
N	Men	Women	Men	Women
	n = 438	n = 360	n = 959	n = 412
	Mean (SD)	Mean (SD)	Mean (SD)	Mean (SD)
Age	50.4 (8.6)	47.2 (7.9)	20.8 (1.1)	21.0 (1.2)
SLI^1^	24.5 (6.1)	24.2 (6.8)	18.6 (4.2)	17.7 (4.5)
METs	38.2 (5.7)	35.3 (5.2)	40.1 (6.5)	36.6 (5.3)
Energy intake	2694 (827)	2027 (568)	3288 (1129)	2066 (627)
Weight (kg)	66.9 (10.9)	61.7 (11.2)	54.8 (8.7)	44.4 (7.4)
Height(cm)	165.0 (6.2)	152.3 (5.7)	166.7 (6.3)	152.6 (5.3)
BMI kg/m^2^	24.6 (3.6)	26.5 (4.3)	19.7 (2.8)	19.0 (2.9)
WHR	0.93 (0.06)	0.83 (0.06)	0.82 (0.04)	0.76 (0.05)
Waist circumference	87.9 (9.4)	83.1 (10.0)	69.9 (6.9)	64.1 (6.8)
Body fat % (skinfolds)	26.6 (5.3)	35.0 (4.5)	13.4 (4.6)	23.0 (5.5)
DXA Total fat (g)^2^	16537 (5364)	23405 (6492)	8776 (4019)	12751 (4244)
DXA L1L4 fat (g)^2^	2449 (983)	2764 (1051)	853 (531)	1003 (534)
Insulin (mu/l)^1,3^	5.8 (3.3, 8.55)	6.15 (3.9, 10)	3.8 (2.9, 5.2)	4.0 (2.8, 5.7)
Glucose mmol/L^1,3^	5.09 (4.72, 5.61)	5.23 (4.81, 5.84)	4.78 (4.47, 5.12)	4.75 (4.47, 5.01)
Triglycerides mmol/L^1^				
Cholesterol mmol/L^1^	4.8 (1.0)	4.9 (1.1)	4.0 (0.9)	4.0 (0.9)
SBP (mm Hg)^1^	122.2 (14.8)	115.4 (14.5)	117.3 (9.2)	109.5 (9.3)
DBP (mm Hg)^1^	80.8 (9.6)	77.9 (9.6)	69.6 (7.7)	68.6 (8.0)
	**N(%)**	**N(%)**	**N(%)**	**N(%)**
Smoking				
Never	342 (78%)	360 (100%)	837 (87%)	412 (100%)
Former/current	96 (22%)	0	122 (13%)	0
Alcohol (drinks/day)				
None	166 (38%)	295 (82%)	263 (27%)	363 (88%)
0 < 1	203 (46%)	61 (17%)	509 (53%)	46 (11%)
1 < 4	57 (13%)	4 (1%)	127 (13%)	2 (0.5%)
≥4	12 (3%)	0	60 (6%)	1 (0.2%)

^1^Some missing data.

^2^Geometric mean and 95% CI are presented for APCAPS subjects.

^3^Glucose and insulin exclude known diabetics and non fasters.

The correlation coefficients between the different measures of adiposity were high in both the IMS and APCAPS groups, generally exceeding 0.7, with the exception of WHR (Additional file 1: Tables S1 and S2).

Measures of adiposity were generally associated with the blood based measures of cardiometabolic risk factors, excepting fasting glucose (Table 2). There was no evidence that the DXA measures of total body fat were more strongly associated with insulin, glucose, triglycerides, or cholesterol in comparison to BMI or body fat estimated through skinfolds in APCAPS (p for heterogeneity all >0.46) or IMS participants. Furthermore, abdominal measures of adiposity (WHR, WC or DXA L1-L4) did not demonstrate stronger associations with any of the blood based cardiometabolic risk factors than whole body measures of adiposity. All these associations were largely unattenuated after multivariable adjustment (data not shown). One quarter (25%, 95% CI 22-28%) of the IMS sample had diabetes, of whom 71% were known diabetics who did not undertake the oral glucose tolerance test. We therefore did not analyse post-load glucose or insulin levels in relation to anthropometric characteristics.

**Table 2:** **Linear regressions showing adiposity in relation to blood-based cardiometabolic risk factors**

	APCAPS		IMS	
	Age and sex adjusted Coefficient (95% CI)	***P***-value	Age and sex adjusted Coefficient (95% CI)	***P***-value
**Insulin** ^**a,b**^	**N = 1,325**		**N = 638**	
DXA Total fat (g)	1.20 (1.16, 1.24)	<0.001	1.27 (1.21, 1.33)	<0.001
BMI kg/m^2^	1.19 (1.15, 1.22)	<0.001	1.29 (1.23, 1.35)	<0.001
Body fat % (skinfolds)	1.18 (1.14, 1.21)	<0.001	1.28 (1.22, 1.33)	<0.001
DXA L1L4 fat (g)	1.18 (1.15, 1.22)	<0.001	1.30 (1.24, 1.36)	<0.001
WHR	1.09 (1.05, 1.12)	<0.001	1.21 (1.15, 1.28)	<0.001
Waist circumference	1.17 (1.14, 1.21)	<0.001	1.31 (1.25, 1.37)	<0.001
**Fasting Glucose** ^**a,b**^	**N = 1,338**		**N = 650**	
DXA Total fat (g)	1.00 (0.99, 1.01)	0.93	1.02 (1.00, 1.03)	0.05
BMI kg/m^2^	1.00 (0.99, 1.00)	0.78	1.02 (1.001, 1.03)	0.04
Body fat % (skinfolds)	1.00 (0.99, 1.01)	0.97	1.02 (1.01, 1.04)	0.001
DXA L1L4 fat (g)	1.00 (1.00, 1.01)	0.73	1.02 (1.01, 1.04)	0.008
WHR	1.00 (0.99, 1.00)	0.29	1.03 (1.02, 1.05)	<0.001
Waist circumference	1.00 (0.99, 1.01)	0.96	1.03 (1.01, 1.04)	0.001
**Triglycerides** ^**b**^	**N = 1,371**		**N = 794**	
DXA Total fat (g)	1.09 (1.07, 1.12)	<0.001	1.04 (1.01, 1.07)	0.01
BMI kg/m^2^	1.10 (1.07, 1.12)	<0.001	1.08 (1.05, 1.11)	<0.001
Body fat % (skinfolds)	1.10 (1.08, 1.13)	<0.001	1.07 (1.04, 1.11)	<0.001
DXA L1L4 fat (g)	1.10 (1.08, 1.13)	<0.001	1.08 (1.04, 1.11)	<0.001
WHR	1.08 (1.06, 1.11)	<0.001	1.11 (1.07, 1.15)	<0.001
Waist circumference	1.10 (1.08, 1.12)	<0.001	1.11 (1.08, 1.14)	<0.001
**Cholesterol**	**N = 1,371**		**N = 794**	
DXA Total fat (g)	0.20 (0.15, 0.25)	<0.001	0.07 (-0.005, 0.14)	0.07
BMI kg/m^2^	0.19 (0.14, 0.24)	<0.001	0.07 (-0.005, 0.14)	0.07
Body fat % (skinfolds)	0.19 (0.14, 0.24)	<0.001	0.12 (0.04, 0.20)	0.004
DXA L1L4 fat (g)	0.21 (0.16, 0.26)	<0.001	0.12 (0.05, 0.19)	0.001
WHR	0.13 (0.09, 0.18)	<0.001	0.13 (0.05, 0.21)	0.001
Waist circumference	0.20 (0.15, 0.24)	<0.001	0.12 (0.05, 0.19)	0.001

Values Are Relative Increases in Biomarkers Per 1 Standard Deviation in Adiposity Measure.

^**a**^Excludes individuals who were diabetic or not fasting.

^b^Outcome measures are log transformed so coefficients represent ratios of geometric means.

Measures of adiposity were also generally associated with blood pressure (Table 3). There was no evidence that the association with blood pressure measures were stronger for DXA measures of total body fat as compared to BMI, or skinfolds. Among IMS participants, the abdominal measures of adiposity, in particular WHR, tended to be more strongly related to SBP than the total body measures of adiposity but there was no strong statistical evidence for these differences (p all ≥0.06). This was not apparent among the APCAPS participants.

**Table 3:** **Linear regressions showing adiposity in relation to blood pressure**

	APCAPS		IMS	
	Age and sex adjusted Coefficient (95% CI)	***P***-value	Age and sex adjusted Coefficient (95% CI)	***P***-value
**SBP**	**N = 1,371**		**N = 644**	
DXA Total fat (g)	1.41 (0.91, 1.91)	<0.001	1.01 (-0.23, 2.24)	0.11
BMI kg/m^2^	2.14 (1.57, 2.72)	<0.001	1.59 (0.35, 2.83)	0.006
Body fat % (skinfolds)	1.72 (1.18, 2.26)	<0.001	1.01 (-0.36, 2.40)	0.15
DXA L1L4 fat (g)	1.75 (1.24, 2.25)	<0.001	1.69 (0.48, 2.89)	0.006
WHR	0.86 (0.24, 1.48)	0.007	2.66 (1.45, 3.88)	<0.001
Waist circumference	2.16 (1.58, 2.74)	<0.001	2.33 (1.08, 3.58)	<0.001
**DBP**	**N = 1,371**		**N = 644**	
DXA Total fat (g)	2.71 (2.31, 3.11)	<0.001	2.42 (1.62, 3.22)	<0.001
BMI kg/m^2^	2.58 (2.18, 2.97)	<0.001	2.52 (1.72, 3.34)	<0.001
Body fat % (skinfolds)	2.65 (2.23, 3.07)	<0.001	2.09 (1.18, 3.00)	<0.001
DXA L1L4 fat (g)	2.82 (2.41, 3.23)	<0.001	2.82 (2.03, 3.60)	<0.001
WHR	1.05 (0.60, 1.52)	<0.001	2.18 (1.36, 3.00)	<0.001
Waist circumference	2.67 (2.27, 3.07)	<0.001	2.91 (2.09, 3.74)	<0.001

Values Are Relative Increases in Biomarkers Per 1 Standard Deviation in Adiposity Measure.

DXA measures additionally adjusted for DXA machine, DXA total fat (grams) and DXA L1L4 fat (grams) additionally adjusted for height.

Logistic regression analyses showed that diabetes, insulin resistance and hypertension were associated with all measures of adiposity among the IMS participants (Table 4). For these three outcomes, this association was not stronger for DXA whole body measures of adiposity compared to BMI, or skinfold measures of body fat. There was no clear evidence that abdominal adiposity measures (WHR, WC and DXA) were more closely associated with hypertension or insulin resistance than whole body measures. In contrast, diabetes had a particularly strong relationship with WHR and there was some statistical evidence that this was stronger than the relationship with DXA total fat, DXA L1L4 fat and BMI (p for all ≤ 0.03). These associations were not attenuated after adjustment for confounders. There were only 14 cases with IFG, and so this outcome was not included in the analyses.

**Table 4:** **Logistic regressions showing adiposity in relation to diabetes and insulin resistance within IMS participants**

	Age and sex adjusted odds ratio (95% CI)	Multivariable adjusted odds ratio (95% CI)*
**Diabetes**	**N = 798**	**N = 798**
DXA Total fat (g)**	1.19 (1.02, 1.39)	1.08 (0.90, 1.30)
BMI kg/m^2^	1.42 (1.21, 1.66)	1.36 (1.14, 1.63)
Body fat % (skinfolds)	1.54 (1.28, 1.84)	1.45 (1.18, 1.79)
DXA L1L4 fat (g)**	1.37 (1.17, 1.61)	1.27 (1.06, 1.52)
WHR	1.88 (1.53, 2.31)	1.85 (1.49, 2.30)
Waist circumference	1.67 (1.40, 2.00)	1.58 (1.31, 1.91)
**Insulin resistance**	**N = 581**	**N = 581**
DXA Total fat (g)**	1.96 (1.63, 2.38)	1.89 (1.54, 2.33)
BMI kg/m^2^	2.10 (1.72, 2.55)	2.06 (1.68, 2.54)
Body fat % (skinfolds)	2.23 (1.78, 2.80)	2.14 (1.68, 2.73)
DXA L1L4 fat (g)**	2.13 (1.72, 2.64)	2.06 (1.64, 2.59)
WHR	1.97 (1.59, 2.45)	2.00 (1.60, 2.51)
Waist circumference	2.42 (1.92, 3.05)	2.41 (1.89, 3.08)
**Hypertension**	**N = 798**	**N = 798**
DXA Total fat (g)**	1.43 (1.22, 1.68)	1.39 (1.17, 1.66)
BMI kg/m^2^	1.49 (1.27, 1.75)	1.46 (1.23, 1.74)
Body fat % (skinfolds)	1.47 (1.23, 1.75)	1.46 (1.20, 1.78)
DXA L1L4 fat (g)**	1.45 (1.24, 1.70)	1.41 (1.19, 1.68)
WHR	1.52 (1.29, 1.79)	1.45 (1.22, 1.73)
Waist circumference	1.61 (1.37, 1.89)	1.56 (1.31, 1.86)

*Adjusted for age, sex, smoking, SLI, total METS, alcohol, caloric intake.

**DXA measures additionally adjusted for DXA machine and height.

Further analyses including DXA measures (DXA percent body fat, truncal fat, L2-L4 regions) and weight were similar to other adiposity measures and were not reported (data available on request).

### Sensitivity analyses

There was consistent evidence among APCAPS for significant effect modification by sex of the relationship between cholesterol and adiposity measures: BMI (p = 0.007), WC (p = 0.04), body fat from skinfolds (p = 0.01), DXA adiposity (p = 0.02) and DXA abdominal measures (p = 0.004). There was also evidence that the relationship between SBP and adiposity measures was modified by sex among the APCAPS participants including: BMI (p = 0.002), WHR (p = 0.01), WC (p < 0.001), bodyfat from skinfolds (p = 0.001), DXA adiposity (p < 0.005) and DXA abdominal adiposity (p < 0.001). Stratifying these analyses by gender showed that relationship between cholesterol and SBP with anthropometric characteristics was weaker in females than in males (Table 5). Effect modification was not consistently apparent among IMS participants or for the other measures of cardiometabolic risk.

**Table 5:** **Linear regressions showing adiposity in relation to cardiometabolic risk factors among APCAPS participants stratified by sex**

	Men		Women	
	Age and sex adjusted Coefficient (95% CI)	***P***, value	Age and sex adjusted Coefficient (95% CI)	***P***-value
**Cholesterol**	**N = 959**		**N = 412**	
DXA Total fat (g)	0.24 (0.18, 0.30)	<0.001	0.11 (0.03, 0.19)	0.01
BMI kg/m^2^	0.23 (0.17, 0.29)	<0.001	0.10 (0.03, 0.18)	0.005
Bodyfat (skinfolds)	0.23 (0.17, 0.29)	<0.001	0.10 (0.03, 0.18)	0.01
DXA L1L4 fat (g)	0.25 (0.19, 0.31)	<0.001	0.10 (0.02, 0.18)	0.02
WHR	0.16 (0.10, 0.21)	<0.001	0.07 (-0.01, 0.16)	0.08
Waist circumference	0.23 (0.17, 0.29)	<0.001	0.13 (0.05, 0.20)	0.003
**SBP**	**N = 959**		**N = 412**	
DXA Total fat (g)	2.16 (1.60, 2.73)	<0.001	0.17 (-0.80, 1.15)	0.73
BMI kg/m^2^	2.59 (1.99, 3.19)	<0.001	1.00 (0.07, 1.93)	0.04
Bodyfat (skinfolds)	2.25 (1.70, 2.81)	<0.001	0.39 (-0.58, 1.37)	0.39
DXA L1L4 fat (g)	2.40 (1.83, 2.97)	<0.001	0.38 (-0.59, 1.35)	0.44
WHR	1.30 (0.70, 1.91)	<0.001	-0.31 (-1.47, 0.85)	0.60
Waist circumference	2.72 (2.12, 3.32)	<0.001	0.70 (-0.30, 1.69)	0.17

Values Are Relative Increases in Biomarkers Per 1 Standard Deviation in Adiposity Measure.

DXA measures additionally adjusted for DXA machine, DXA total fat (grams) and DXA L1L4 fat (grams) additionally adjusted for height. Sex specific Z-scores were created for these analyses.

## Discussion

Our study included adults across a broad age spectrum and urban and rural in Hyderabad, India, and assessed a range of blood based markers of cardiometabolic risk and blood pressure as well as conventional and DXA measures of adiposity. We found strong and consistent relationships between different adiposity measures and cardiometabolic risk factors. There was a lack of evidence for better prediction of prevalent cardiometabolic risk factors by DXA measures of adiposity as opposed to BMI, or skinfold measures. There was also no evidence that abdominal measures of adiposity were more closely related to cardiometabolic risk factors, excepting the relationship between WHR and diabetes.

Previous studies suggest that diabetes develops at lower levels of BMI among Asians, including Indians, compared to Whites [10, 11]. The vulnerability of Indians to diabetes and cardiovascular disease may be because of the higher adiposity for a given BMI [12, 14, 16], predisposition towards abdominal adiposity [20, 21], or it may be independent of adiposity, for instance influenced by life course factors [3, 9, 23], or genetic vulnerability [27–29]. This study did not demonstrate that DXA measures, which more accurately assess the amount of body fat, were more predictive of prevalent cardiometabolic risk factors than BMI, or skinfold measures of adiposity. The few comparable data from large population-based studies also fail to show that DXA measures of adiposity are better predictors of markers of diabetes or hypertriglyceridemia than simpler conventional adiposity measures [37–41]. One potential explanation is that BMI presents a composite index of risk – reflecting adiposity, lean mass and height, and while increased weight and shorter stature may contribute towards increasing risk of cardiometabolic risk, increased lean mass (which raises BMI) may protect from cardiometablic risk [42]. This means that BMI may do better than expected as an index of CVD risk because it captures more than just adiposity.

We did not find evidence to suggest mediation of vulnerability through excess abdominal adiposity since markers of cardiometabolic risk, were not more closely associated with abdominal adiposity markers than whole body measures of adiposity. The exception was the stronger relationship between WHR and diabetes than with other adiposity measures. This field remains controversial, as some studies show that abdominal adiposity (e.g. WHR, WC) is more closely related to diabetes and coronary disease than more general obesity measures (e.g. BMI) [20–22], while others do not show this difference [43, 44]. A large review showed that WC was more strongly related than BMI to prevalent diabetes, but did not observe this difference in the Asian studies [45], while other reviews show that central adiposity is more closely related to diabetes prevalence among Asians [10, 46].

The possibility that the vulnerability of people in India to diabetes and its sequelae is unrelated to adiposity, perhaps relating to genetic vulnerability [27–29] was not explored with the current study design.

### Strengths and limitations

A key strength was that both DXA scanning and conventional anthropometry were undertaken. Furthermore, a large age range was included, with participants from both rural and urban areas. The study sample was relatively large, and examined Indian people in India, rather than Indians in other countries. We included comprehensive assessment of blood based cardiometabolic risk factors and blood pressure, but not metabolic syndrome. Limitations include the cross-sectional design and relatively low response rate, which raises the possibility of selection bias as some differences in characteristics were noted between responders and non-responders. There may also be limited generalisability of the results to the Indian population since the IMS participants were sampled from factories and so were more affluent and more likely to be in stable employment than the Indian population in general. DXA cannot distinguish between visceral and subcutaneous fat, which may confer different health risks. The cut-off used for determination of outcomes were mostly based on Western populations, which may not always be appropriate for an Indian population. Additionally, the Durnin and Womersley formula for calculation of body fat from skinfolds [33], may not be appropriate for this ethnic group.

## Conclusions

We did not demonstrate that DXA measures were better predictors of prevalent cardiometabolic risk factors than conventional measures of adiposity. This study found evidence that WHR is more strongly associated with markers of diabetes than total body adiposity.

## Electronic supplementary material

Additional file 1: Table S1: Correlation Coefficients Between Anthropometric Measures: Males. **Table S2.** Correlation Coefficients Between Anthropometric Measures: Females. (DOCX 15 KB)

## Abbreviations

AIIMS: All India Institute of Medical Sciences
APCAPS: Andhra Pradesh Children and Parent Study
BMI: Body mass index
CVD: Cardiovascular disease
DBP: Diastolic blood pressure
DXA: Dual-energy X-ray absorptiometry
HNT: Hyderabad Nutrition Trial
HOMA: Homeostasis model assessment
IFG: Impaired fasting glucose
IMS: Indian Migration Study
MET: Metabolic equivalent tasks
SBP: Systolic blood pressure
SLI: Standard of living index
WC: Waist circumference
WHR: Waist hip ratio.
