# Supplementary material for: Is vulnerability to cardiometabolic disease in Indians mediated by abdominal adiposity or higher body adiposity
Source: BMC Public Health. 2014 Dec 1;14:1239. doi: 10.1186/1471-2458-14-1239 (PMC4289237; doi:10.1186/1471-2458-14-1239)
Supplement: Supplementary file 1 — Additional file 1: Table S1: Correlation Coefficients Between Anthropometric Measures: Males. Table S2. Correlation Coefficients Between Anthropometric Measures: Females. (DOCX 15 KB) [file 12889_2014_7348_MOESM1_ESM.docx]

Additional file 1

**Web table 1: Correlation Coefficients Between Anthropometric Measures: Males**

|  | BMI | WHR | WC | DXA Total fat | DXA L1L4 fat | Body fat % (skinfolds) |
| --- | --- | --- | --- | --- | --- | --- |
| IMS | N=438 |  |  |  |  |  |
| BMI | 1.00 |  |  |  |  |  |
| WHR | 0.48 | 1.00 |  |  |  |  |
| WC | 0.89 | 0.74 | 1.00 |  |  |  |
| DXA total fat | 0.89 | 0.40 | 0.86 | 1.00 |  |  |
| DXA L1L4 fat | 0.87 | 0.52 | 0.89 | 0.95 | 1.00 |  |
| Body fat % (skinfolds) | 0.77 | 0.44 | 0.74 | 0.77 | 0.74 | 1.00 |
|  |  |  |  |  |  |  |
| APCAPS | N=959 |  |  |  |  |  |
| BMI | 1.00 |  |  |  |  |  |
| WHR | 0.54 | 1.00 |  |  |  |  |
| WC | 0.91 | 0.72 | 1.00 |  |  |  |
| DXA total fat | 0.88 | 0.47 | 0.86 | 1.00 |  |  |
| DXA L1L4 fat | 0.86 | 0.54 | 0.87 | 0.96 | 1.00 |  |
| Body fat % (skinfolds) | 0.83 | 0.44 | 0.79 | 0.90 | 0.86 | 1.00 |

**Web table 2: Correlation Coefficients Between Anthropometric Measures: Females**

|  | BMI | WHR | WC | DXA Total fat | DXA L1L4 fat | Body fat % (skinfolds) |
| --- | --- | --- | --- | --- | --- | --- |
| IMS | N=360 |  |  |  |  |  |
| BMI | 1.00 |  |  |  |  |  |
| WHR | 0.25 | 1.00 |  |  |  |  |
| WC | 0.85 | 0.64 | 1.00 |  |  |  |
| DXA total fat | 0.92 | 0.15 | 0.81 | 1.00 |  |  |
| DXA L1L4 fat | 0.86 | 0.35 | 0.86 | 0.89 | 1.00 |  |
| Body fat % (skinfolds)) | 0.73 | 0.27 | 0.68 | 0.74 | 0.69 | 1.00 |
|  |  |  |  |  |  |  |
| APCAPS | N=412 |  |  |  |  |  |
| BMI | 1.00 |  |  |  |  |  |
| WHR | 0.39 | 1.00 |  |  |  |  |
| WC | 0.87 | 0.71 | 1.00 |  |  |  |
| DXA total fat | 0.90 | 0.29 | 0.82 | 1.00 |  |  |
| DXA L1L4 fat | 0.88 | 0.46 | 0.87 | 0.92 | 1.00 |  |
| Body fat% (Skinfolds) | 0.74 | 0.28 | 0.68 | 0.84 | 0.77 | 1.00 |
